# Prevalence of methicillin-resistant *Staphylococcus aureus* carriage on admission among patients attending regional hospitals in Dar es Salaam, Tanzania

**DOI:** 10.1186/s13104-017-2668-8

**Published:** 2017-08-22

**Authors:** Agricola Joachim, Sabrina J. Moyo, Lillian Nkinda, Mtebe Majigo, Elia Mbaga, Naboth Mbembati, Said Aboud, Eligius F. Lyamuya

**Affiliations:** 1 Department of Microbiology and Immunology, Muhimbili University of Health and Allied Sciences, Dar Es Salaam, Tanzania; 2 Department of Clinical Science, University of Bergen, Bergen, Norway; 3 Department of Epidemiology and Biostatistics, Muhimbili University of Health and Allied Sciences, Dar Es Salaam, Tanzania; 4 Department of Surgery, Muhimbili University of Health and Allied Sciences, Dar Es Salaam, Tanzania

**Keywords:** MRSA nasal carriage, Infection, Antibiotic resistance

## Abstract

**Background:**

Methicillin-resistant *Staphylococcus aureus* (MRSA) is a major pathogen responsible for hospital and community acquired infection. Colonization with MRSA is associated with a high risk of developing infection. This study aimed to determine the rate of MRSA carriage on admission and the associated risk factors among patients attending regional hospitals, in Dar es Salaam, Tanzania.

**Results:**

A total of 258 patients were included in this study. Nasal swabs were collected on admission to the hospital and after 48 h of hospital stay for detection of MRSA. Of 258 patients enrolled, 89 (34.5%) were colonized with *S. aureus* and out them 22 (24.7%) were carriers of MRSA, giving an overall MRSA nasal carriage rate of 8.5% (22/258). One patient acquired MRSA while admitted in the hospital. Most of the *S. aureus* isolates 85 (95.5%) were resistant to penicillin. Resistance to gentamycin, ciprofloxacin, kanamycin, linezolid and mupirocin were 14.6, 11.2, 11.2, 3.4 and 1.1%, respectively. The prevalence of inducible clindamycin resistance, constitutive clindamycin resistance, MS phenotype (resistance to erythromycin alone), and multidrug resistance was 21.3, 3.4, 12.4, and 16.9%, respectively. We observed a statistically significant association between MRSA and multiple drugs resistance among *S. aureus* isolates (p = 0.001). Of the risk factors investigated none were statistically significant associated with MRSA.

**Conclusion:**

There is a high prevalence of MRSA among patients on admission at the two municipal hospitals in Dar es Salaam. The high prevalence of MRSA and the increased rates of resistance to commonly used antimicrobials among MRSA isolates call for attention to the importance of including the screening of MRSA in our hospitals setting in order to prevent further spread of MRSA strains to other patients and to the communities. Control and prevention strategies should be emphasized including decolonization.

## Background

Methicillin-resistant *Staphylococcus aureus* (MRSA) is an important cause of hospital-acquired infection leading to a high morbidity and mortality among patients worldwide. Recent studies have also reported increasing rates of MRSA infections in the community settings, which are referred to as Community acquired MRSA [1, 2]. MRSA remains a major pathogen in nosocomial infections in developing countries [3]. Carriage of antimicrobial-resistant strain like MRSA puts an individual at high risk of developing infection [4]. The rate of developing infection following MRSA colonization is reported to be approximately 30% [4, 5]. Of concern is the high mortality associated with MRSA infections. Infection due to *S. aureus* ranges from mild to moderate skin and soft tissue infections to invasive life-threatening systemic infections [6].

Carriage rates or infections with MRSA vary by geographical location, type of health care facility, and the specific population being studied. Studies have reported prevalence of MRSA carriage on admission ranging from 2.6 to 8% with higher prevalence reported among elderly patients [7–11]. Reports have shown that approximately 80–95% of MRSA carriages are asymptomatic [12]. However, harboring of MRSA strains during the time of hospital admission may have negative consequences not only on patients’ management but also affect the implementation of infection control measures in the hospital especially where resources are limited [3]. One of the consequences of admitted patients who are carrying MRSA is the increase in the risk of transmitting the pathogen to other patients and health workers [13, 14]. Populations at risk of acquiring MRSA infection include people with underlying chronic infections [15–17]. Such conditions may result in severe complications and fatal consequences, especially if multiple antibiotic resistant strains are involved. Other reported risk factors for MRSA acquisition include prolonged hospitalization, visiting an outpatient clinic, patients with a skin or soft-tissue infection, working in health care facilities as well as history of antibiotic use [17–20]. However, the risk factors for MRSA colonization at the time of hospital admission among patients are not well known. Identifying MRSA colonization at admission could target a high-risk population that may benefit from interventions to decrease the risk for developing MRSA infection.

Studies have shown that strategies to screen for colonized patients at admission and decolonize them may lead to the reduction of the transmission rate of MRSA [21, 22]. Elimination of MRSA carriage through the application of decolonization agents, such as nasal mupirocin and chlorhexidine soap has been reported elsewhere [14].

Development of resistance to antimicrobial agents among *staphylococci* is an increasing problem worldwide [23]. The increasing rate of MRSA multiple-drug resistant strains, which limits the therapeutic options available for the management of MRSA related infections, has become a serious concern worldwide. Several studies have reported the use of macrolide-lincosamide-streptogramin B (MLS_B_) in the treatment of staphylococcal infections, where clindamycin is used as alternative treatment. However risks of treatment failure due MLS_B_ inducible resistance are reported to frequently increase [24–26].

Previous studies conducted in Tanzania at Muhimbili national hospital (MNH) and Bugando medical center have documented an increasing prevalence of hospital-acquired MRSA; 0.4% [27], 8% [28], 16.3% [29] and 23% [30]. These studies used clinical specimens from hospitalized patients who presented with symptoms and/or signs of infection. In Tanzania, no study has been conducted on MRSA screening among patients at the time of admission to hospitals. The current study was undertaken to investigate the rate of MRSA carriage on admission and the associated risk factors among patients attending the Amana and Mwananyamala regional hospitals in Dar es Salaam, Tanzania.

## Methods

### Study design and sampling procedures

This was a hospital based cross-sectional study conducted at two regional hospitals, Mwananyamala and Amana in Dar es Salaam, Tanzania. Using Kish–Leslie formula and a reported prevalence of MRSA of 23% at MNH, Dar es Salaam Tanzania by Moyo et al. [30], a minimum sample size of 283 was targeted. However, were able to enroll 258 patients. All eligible patients admitted at emergency department or medical ward between March and August 2015 were recruited in the study after obtained an informed consent. Children below 5 years of age and patients who were using antibiotic at the time of recruitment or within 2 weeks were excluded. Structured questionnaire was used to collect social demographic information including age, sex, level of education and residence. Risk factors associated with MRSA including current and previous medical history and use of antibiotic in the past 3 months were also collected. Furthermore, patients who stayed in the hospital for 48 h or more following admission were requested to provide a second set of nasal specimen. These were the same patients whom we collected nasal samples on admission and were more likely to have serious illness compared to other patients. A total of 20 patients were available for second sample collection.

### Sample collection and transportation

Well-trained health personnel collected nasal specimens from both anterior nares of each patient using a sterile cotton wool swab on admission. Second set of nasal swab was collected 48–72 h after admission. Nasal swabs were placed in Stuart transport media and transported to the microbiology and immunology laboratory at Muhimbili University of Health and Allied Sciences (MUHAS) and processed within 24 h of collection.

### Laboratory procedures

Nasal swabs were inoculated into mannitol salt agar plates (OXOID, Basingstoke, United Kingdom) for *S. aureus* isolation. The plates were incubated at 37 °C and examined for growth after 24–48 h. Isolates were identified as *S. aureus* based on colonial morphology, gram staining, catalase test, coagulase test and DNase test positive. The antimicrobial susceptibility testing was carried out using Kirby–Bauer’s disc diffusion method according to clinical and laboratory standards institute (CLSI) 2015 guidelines [31]. The following standard antibiotic disks (OXOID UK) were used; penicillin G (10 U), kanamycin (30 µg), gentamicin (10 µg), erythromycin (15 µg), clindamycin (2 µg), ciprofloxacin (5 µg), linezolid (30 µg) and mupirocin (5 µg). A standard inoculum was prepared by direct colony suspension in saline and compared with 0.5 McFarland standard turbidity and inoculated on Muller Hinton agar plate (OXOID UK). Plates were incubated at 35 °C for 18–24 h. Results were interpreted according to the CLSI guidelines [31]. MRSA detection was done using cefoxitin discs (OXOID UK) according to CLSI 2015 guidelines. All isolates resistant to cefoxitin were considered as MRSA. An inhibition zone of 21 mm or less around cefoxitin disc indicated MRSA [31]. *S. aureus* ATCC 25923 was used for quality control.

In addition clindamycin inducible resistance was also tested by D test as per CLSI guidelines [31]. Briefly, erythromycin (15 μg) disk was placed at a distance of 15–26 mm (edge to edge) from clindamycin (2 μg) disk on a Mueller–Hinton agar plate. After overnight incubation, plates were examined for the formation of flattened zone of inhibition adjacent to the erythromycin disk. Formation of D-shape with erythromycin indicated a positive clindamycin inducible resistant (iMLS_B_). Resistance to both clindamycin and erythromycin was recorded as constitutive resistance (cMLS_B_) and MS phenotype if the isolate was resistant to erythromycin only [31].

### Data analysis

Data obtained were analysed using statistical program for social sciences (SPSS) version 17.0. Chi square test or Fisher’s exact test was used where applicable to compare the proportions of categorical independent and dependent variables. Univariate and multivariate analysis were performed to determine the risk factors associated with nasal *S. aureus* and MRSA colonization. A *p* value of <0.05 was considered as statistically significant.

## Results

A total of 258 patients were enrolled during the study period. Of these, 150 (58.1%) were females. The mean age was 34 years, ranging from 10 to 80 years. Half of the patients 129 (50%) were from Kinondoni, 97 (38%) from Ilala and 32 (12%) from Temeke districts. Majority of the patients 133 (51.6%) had informal or attained primary education. Of 258 patients, 20 (7.8%) had history of previous hospitalization while 60 (23%) had attended outpatient clinic prior to the current admission. Thirty-seven (14.3%) patients had received antibiotics within the past 3 months.

### Nasal carriage of *S. aureus*, MRSA, and antibiotic susceptibility patterns

A total of 89/258 (34.5%) *S. aureus* were isolated from the samples collected on admission to the hospital. Of the 89 isolates, 22 (24.7%) were MRSA while 67 (75.3%) were methicillin susceptible *S. aureus* (MSSA) making the overall prevalence of MRSA among all patients to be 22/258 (8.5%). Only 20 patients were available for second samples collection due to the fact that most of the patients were discharged from the hospital before 48 h of admission. Of the 20 samples collected, four had *S. aureus* isolated, and two out of these had MRSA carriage. One patient had MRSA on admission while the second one was MRSA negative on admission. Table 1 shows the characteristics of the patients at admission to the hospital, according to their MRSA status (positive or negative). Of the total number of patients colonized with MRSA, more than half 12 (54.5%) were male, half 11(50%) were aged 18–30 years and 13 (59.1%) had informal or primary education level. Most of patients who tested MRSA positive 21 (95.5%) had no history of previous hospital admission and 18 (81.8%) had neither history of attending outpatient clinic nor use of antibiotic in the past 3 months. A higher frequency of MRSA was detected among patients diagnosed with acute illness 17 (77.3%) on admission compared to patients with chronic illness 5 (22.7%) Table 1.

**Table 1:** Characteristics of study participants with and without MRSA

Characteristic	MRSA positive	MRSA negative
	N = 22	N = 236
	n (%)	n (%)
Age group in years		
7–17	3 (13.6)	21 (8.9)
18–30	11 (50)	91 (38.6)
31–60	7 (31.8)	102 (43.2)
>60	1 (4.5)	22 (9.3)
Sex		
Male	12 (54.5)	96 (40.7)
Female	10 (45.5)	140 (59.3)
Education attained		
Primary education and below	13 (59.1)	120 (50.8)
Secondary education and above	9 (40.9)	116 (49.2)
History of antibiotic use		
Yes	4 (18.2)	33 (14.0)
No	18 (81.8)	203 (86.0)
History of hospitalisation		
Yes	1 (4.5)	19 (8.1)
No	21 (95.5)	217 (91.9)
History of attending OPC		
Yes	4 (18.2)	56 (23.7)
No	18 (81.8)	180 (76.3)
Type of illness on admission		
Chronic illness	5 (22.7)	39 (16.5)
Acute illness	17 (77.3)	197 (83.5)

*OPC* outpatient clinic

Most of the *S. aureus* isolates 85 (95.5%) were resistant to penicillin. Resistance to gentamycin, ciprofloxacin, kanamycin and linezolid were 14.6, 11.2, 11.2 and 3.4%, respectively. Only one (1.1%) isolate that was MSSA was found to be resistant to mupirocin. Antimicrobial resistance pattern of MRSA and MSSA are summarized in Table 2. We found higher rates of resistance to gentamycin, ciprofloxacin and kanamycin among MRSA isolates compared to MSSA isolates, (p = 0.00).

**Table 2:** Antimicrobial resistance pattern among MRSA and MSSA isolates

Antimicrobial drug	MRSA	MSSA	p value
	n = 22 (%)	n = 67 (%)	
Penicillin	NA	63 (94)	
Ceftriaxone	NA	0	
Gentamycin	10 (45.5)	3 (4.5)	0.00
Ciprofloxacin	8 (36.4)	2 (3)	0.00
Kanamycin	8 (36.4)	2 (3)	0.00
Linezolid	2 (9.1)	1 (1.5)	0.23
Mupirocin	0 (0)	1 (1.5)	

*MRSA* methicillin-resistant *Staphylococcus aureus*, *MSSA* methicillin-susceptible* S. aureus*, *NA* not applicable

The prevalence of iMLS_B_, cMLS_B_, MS phenotype, and MDR was 21.3, 3.4, 12.4, and 16.9%, respectively. There was a statistically significant association between MRSA and multiple drugs resistance (MDR) among *S. aureus* isolates (p = 0.001) (Table 3).

**Table 3:** Prevalence of different antimicrobial resistance type among MRSA and MSSA isolates

Resistance type	Overall N = 89	MRSA N = 22	MSSA N = 67	p value
	n (%)	n (%)	n (%)	
iMLS_B_	19 (21.3)	7 (31.8)	12 (17.9)	0.22
cMLS_B_	3 (3.4)	2 (9.1)	1 (1.5)	0.14
MS phenotype	11 (12.4)	6 (27.3)	5 (7.5)	0.02
MDR	19 (21.3)	16 (72.7)	3 (4.5%)	0.001

### Factors associated with MRSA carriage

Chronic illness increases the risk of MRSA colonization two times compared to acute illness (odd ratio OR, 1.96 [95% CI 0.52–7.31]). Male patients are more likely to be MRSA carrier than females with an odds ratio 2.15 [95% CI 0.81–5.72]. The use of antibiotic within the past 3 months appears to influence the risk of MRSA carriage (OR 1.36 [95% CI 0.43–4.20]) whereas as history of previous hospitalization or attending outpatient clinic did not influence the rate of MRSA colonization (OR, 0.71 [95% CI 0.07–6.45] and 0.88 [95% CI 0.25–3.10]), respectively (Table 4).

**Table 4:** Univariate and multivariate association between MRSA carriage and risk factors

Characteristic	N = 258	MRSA positive n (%)	Univariate p value; OR (95%)	Multivariate p value; OR (95%)
Age group in years				
7–17	24	3 (12.5)	0.29; 3.2 (0.37–40.5)	0.25; 4.1 (0.36–47.6)
18–30	102	11 (10.8)	0.178; 4.6 (0.49–44.5)	0.126; 6.1 (0.60–63.6)
31–60	109	7 (3.6)	0.99; 1.5 (−0.57–3.70)	0.88; 1.2 (0.11–12.7)
>60	23	1 (4.3)	1	1
Sex				
Male	108	12 (11.1)	0.124; 2.15 (0.81–5.72)	0.104; 2.3 (0.84–6.42)
Female	150	10 (6.7)	1	1
Education attained				
Primary education and below	133	13 (9.8)	0.46; 1.39 (0.57–3.39)	0.36; 1.6 (0.57–4.46)
Secondary education and above	125	9 (7.2)	1	1
History of antibiotic use				
Yes	37	4 (11.0)	0.59; 1.36 (0.43–4.2)	0.38; 1.72 (0.5–5.8)
No	221	18 (8.0)	1	1
History of hospitalisation				
Yes	20	1 (5.0)	0.76; 0.715 (0.079–6.45)	0.66; 0.60 (0.63–5.85)
No	238	21 (8.8)	1	1
History of attending OPC				
Yes	60	4 (6.7)	0.84; 0.88 (0.25–3.1)	0.92; 0.94 (0.26–3.33)
No	198	18 (9.1)	1	1
Type of illness on admission				
Chronic illness	44	5 (11.4)	0.314; 1.96 (0.52–7.31)	0.41; 1.74 (0.46–6.57)
Acute illness	214	17 (7.9)	1	

*OPC* outpatient clinic

## Discussion

The present study determined the rate of MRSA carriage on admission among patients attending hospitals in Dar es Salaam. The overall prevalence of MRSA carriage among all patients investigated in this study was 8.5%. These findings are consistent with reports from other studies [7, 8, 11]. We observed a high proportion of MRSA (24.7%) among patients who were colonized with *S. aureus*. The prevalence reported here is comparable with reports from previous studies conducted in Tanzania [30] but higher than the prevalence reported by Mshana et al. who found a prevalence of 16.2% [29]. The higher prevalence in our study could be due to differences in the populations studied. While this study was looking for MRSA carriage among admitted patients the other study [29] searched for MRSA from clinical isolates. Differences observed could also be due to different geographical locations of these studies. Our study was conducted in Dar es Salaam, which is considered to be more overcrowded/overpopulated city; this might have increased the risk of transmission.

Previous study has demonstrated that the risk of acquiring MRSA increase with the length of hospital stay [32]. In this study we also aimed to assess the risk of acquiring MRSA for those who were initially free of the organism at the time of admission but acquired the same while in the hospital. Twenty patients were available for second samples collection, 48 h after admission. One patient who was MRSA negative at the time of admission was MRSA positive after 48 h of staying in the hospital indicating that the organism was acquired while in the hospital.

The antimicrobial resistance pattern reported in this study shows that MRSA isolates were resistant to most commonly used antibiotics. Resistance to gentamycin, ciprofloxacin and kanamycin were significantly higher among MRSA isolates compared to MSSA. Low resistance towards Linezolid (3.4%) indicates that this antibiotic might be an option for empirical therapy of MRSA infections at our hospitals. All MRSA isolates were sensitive to mupirocin with only one MSSA isolate (1.1%) demonstrating resistance. Various rates of mupirocin resistance among MRSA isolates have been described in hospitalized patients ranging from 0 to 65% [33–37]. Our results indicate that mupirocin is still suitable for decolonization as well as treatment of staphylococcal skin infection in our settings.

The use of macrolide-lincosamide-streptogramin B (MLSB) antibiotics in the treatment of both methicillin susceptible and resistant staphylococcal infections with clindamycin being used as alternative treatment has been reported [24]. In the current study we observed high prevalence of iMLSB in both MRSA and MSSA isolates. Similar observations have been reported by other studies conducted in and outside our settings [23, 26, 29, 30]. The high prevalence of iMLSB is an indication of possible therapeutic failure when using clindamycin in *S. aureus* infection. Notably therapeutic failures caused by macrolide-lincosamide-streptogramin B inducible resistance are being more commonly reported [26]. A limitation of this study is lack of molecular confirmation and characterization of MRSA strains due to financial constraints making is difficult to determine the circulating MRSA genotypes.

In the current study we found patients with chronic illness had twofold increase in the risk of acquiring MRSA compared to patients with acute illness. Similar findings have been reported earlier [16, 17]. This can be due to the fact that most of the patients with chronic disease visit hospital often and thus increasing the chance of acquiring the pathogen. Previous studies have reported exposure to antibiotic is associated with risk of MRSA colonization [18, 38]. Our findings showed a trend of but non-significantly higher MRSA among patients with previous exposure to antibiotics. This could be due to our small sample size. Alternatively, there is a possibility that some of the patients may have not recalled properly the information on antibiotic use for the past 3 months and even for those who reported some could not mention the name of antibiotic or type of drug used thus underestimating the role of this factor as risk for MRSA acquisition. Furthermore, our findings differ from the findings of other studies, which have reported history of previous hospitalization to be associated with increased risk of MRSA carriage [18, 38]. The lack of association may be due to the small number of patients with such risk in the population investigated resulting into lack of power to identify such associations, and this is another limitation of our study. Studies conducted elsewhere have strongly suggested that males have a higher risk of MRSA carriage [39, 40]. In this study, we observed that the risk of acquiring MRSA strain were twice higher in male than in the female patients. This could be attributed by gender differences in behavior practices and hygiene such as hand washing and use of soap or playing contact sports and occupation, which may influence MRSA colonization. Other risk factors associated with MRSA colonization have been reported elsewhere [9, 11, 41] but we found no association with age or level of education.

## Conclusion

We report a high prevalence of MRSA among patients on admission at the two municipal hospitals in Dar es Salaam, Tanzania. The high prevalence of MRSA and the increased rates of resistance to commonly used antimicrobials among MRSA isolates call for attention to the importance of including the screening of MRSA in our hospitals setting in order to prevent further spread of MRSA strains to other patients and to the communities. Control and prevention strategies should be emphasized including decolonization of careers.

## Abbreviations

CLSI: clinical and laboratory standards institute guidelines
MUHAS: Muhimbili University of Health and Allied Sciences
MPL: microbiology pathology laboratory
MRSA: methicillin resistant *Staphylococcus aureus*
MSSA: methicillin-susceptible S. aureus
MLS_B_: macrolide-lincosamide-streptogramin B
cMLS_B_: constitutive resistance
iMLS_B_: clindamycin inducible resistance
