# Erratum to: Prevalence of methicillin-resistant *Staphylococcus aureus* carriage on admission among patients attending regional hospitals in Dar es Salaam, Tanzania

**DOI:** 10.1186/s13104-017-2812-5

**Published:** 2017-09-19

**Authors:** Agricola Joachim, Sabrina J. Moyo, Lillian Nkinda, Mtebe Majigo, Elia Mmbaga, Naboth Mbembati, Said Aboud, Eligius F. Lyamuya

**Affiliations:** 1 Department of Microbiology and Immunology, Muhimbili University of Health and Allied Sciences, Dar Es Salaam, Tanzania; 2 Department of Clinical Science, University of Bergen, Bergen, Norway; 3 Department of Epidemiology and Biostatistics, Muhimbili University of Health and Allied Sciences, Dar Es Salaam, Tanzania; 4 Department of Surgery, Muhimbili University of Health and Allied Sciences, Dar Es Salaam, Tanzania

## Erratum to: BMC Res Notes (2017) 10:417 DOI 10.1186/s13104-017-2668-8

Following publication of the original article [1], author Elia Mmbaga pointed out that her name had been misspelt as Elia Mbaga.

The original article has been corrected.

## References

[1] Joachim A, Moyo S, Nkinda L, Majigo M, Mmbaga E, Mbembati N, Aboud S, Lyamuya EF (2017). Prevalence of methicillin-resistant *Staphylococcus aureus* carriage on admission among patients attending regional hospitals in Dar es Salaam, Tanzania. BMC Res Notes.

